# Ratio-Dependent Regulation of Butyrate Production by 2′-Fucosyllactose and Fructooligosaccharides in a *Bifidobacterium bifidum*-*Faecalibacterium prausnitzii* Co-Culture

**DOI:** 10.3390/microorganisms14081812

**Published:** 2026-08-17

**Authors:** Sotaro Iguchi, Haruka Onodera, Yosuke Komatsu, Takeshi Kokubo

**Affiliations:** Institute of Health Science, Kirin Holdings Co., Ltd., 2-26-1-12-12 Muraoka-Higashi, Fujisawa 255-8555, Japan; sotaro_iguchi@kirin.co.jp (S.I.); haruka_onodera@kirin.co.jp (H.O.); yosuke_komatsu@kirin.co.jp (Y.K.)

**Keywords:** 2′-fucosyllactose, human milk oligosaccharides, fructooligosaccharides, *Bifidobacterium bifidum*, *Faecalibacterium prausnitzii*, substrate complementarity, butyrate

## Abstract

An appropriate 2′-fucosyllactose (2′-FL) and fructooligosaccharides (FOS) mixing ratio may enhance butyrate production through substrate complementarity and metabolite-mediated interactions; however, the substrate balance required for this effect remains unclear. This study investigated how different 2′-FL/FOS ratios affect butyrate production in a two-species co-culture of *Bifidobacterium bifidum* and *Faecalibacterium prausnitzii*. 2′-FL and FOS were mixed at different weight ratios and added to monocultures and two-species co-cultures. The bacterial growth, relative abundance, and organic acid production were also evaluated. In monoculture, *F. prausnitzii* grew under FOS-containing conditions, and *B. bifidum* growth increased as the proportion of 2′-FL increased. In the co-culture of *F. prausnitzii* and *B. bifidum*, total OD_600_ and the production of lactate and acetate generally increased under 2′-FL-rich conditions. In contrast, butyrate production showed a nonlinear response and peaked at a 2′-FL/FOS ratio of 10:90 (*w*/*w*), although total co-culture growth was not maximized under this condition. These findings suggest that butyrate production in this co-culture system is regulated in a ratio-dependent manner and is not solely explained by total bacterial growth or acetate accumulation. Rather, the observed butyrate profile likely reflected the combined effects of substrate complementarity, maintenance of the *F. prausnitzii* population, and metabolite exchange between the two species.

## 1. Introduction

Human milk oligosaccharides (HMOs) are characteristic components of human breast milk and are recognized as bioactive constituents involved in shaping infant gut microbiota and supporting immune development [1,2,3]. Among them, 2′-fucosyllactose (2′-FL), a fucosylated trisaccharide composed of glucose, galactose, and fucose, is one of the most prevalent and abundant HMOs in human milk [4]. Recent advances in production technologies based on microbial fermentation and enzymatic synthesis have enabled industrial-scale production of 2′-FL [5]. Clinical studies in formula-fed infants receiving 2′-FL-supplemented formula have reported a shift in gut microbiota toward a profile more similar to that of breastfed infants, accompanied by an increased predominance of bifidobacteria as well as improvements in stool consistency, including softer stools [6,7,8]. In addition to its use in infant formula, 2′-FL has attracted interest as a dietary supplement for adults. However, the clinical benefits of 2′-FL intake in adults remain unclear, and current evidence mainly indicates changes in gut microbial composition [9].

Against this background, clarifying the value of 2′-FL in adults and facilitating its practical application require assessing both gut microbiota composition and microbiota-derived metabolites. Butyrate, a major short-chain fatty acid (SCFA), serves as the principal energy source for colonocytes and is involved in maintaining intestinal barrier function and regulating inflammation [10,11,12]. Thus, butyrate is an important metabolite in adult intestinal physiology. Butyrate is primarily produced by the anaerobic microorganisms in the gut. *Faecalibacterium prausnitzii* (*F. prausnitzii*) is consistently regarded as one of the major butyrate-producing bacteria in the human gut and is frequently detected in healthy individuals [13]. Therefore, promoting the metabolic activity of butyrate-producing bacteria, including *F. prausnitzii*, may be a rational strategy for evaluating and improving intestinal function in adults. Thus, dietary strategies focused on efficiently enhancing butyrate production may help expand the functional relevance of 2′-FL beyond infant nutrition.

2′-FL is utilized by *Bifidobacterium longum* subsp. *infantis* (*B. infantis*) and *Bifidobacterium bifidum* (*B. bifidum*), leading to acetate as a major fermentation product [14,15,16,17]. Acetate can be utilized as a precursor or co-substrate for butyrate production in certain butyrate-producing bacteria [18,19,20,21]. These findings suggest that 2′-FL may indirectly support butyrate production by providing acetate through its utilization by bifidobacteria.

However, butyrate-producing bacteria do not necessarily utilize 2′-FL directly. Phenotypic characterization of multiple *F. prausnitzii* strains has shown that 2′-FL is not utilized by these strains [22]. In addition, studies using various HMOs, including 2′-FL, have indicated that the growth of *F. prausnitzii* may be limited when HMOs serve as substrates [23]. When 2′-FL is used alone, the growth and metabolic activity of acetate-utilizing butyrate-producing bacteria become limiting factors for the stable enhancement of butyrate production.

Fructooligosaccharides (FOS) are promising candidates to overcome this limitation, particularly given their applicability to adult nutrition. FOS are representative prebiotics that are widely used in food and dietary supplements, and can be fermented by the gut microbiota into beneficial metabolites, including SCFAs [24]. Butyrate-producing bacteria, including members of the genera *Faecalibacterium*, *Roseburia*, *Eubacterium*, and *Anaerostipes*, have been identified as key contributors to gut homeostasis [25]. Studies have investigated whether these bacteria can utilize FOS by examining their functional genes [26]. Certain FOS components, such as 1-kestose, have been reported to significantly stimulate *F. prausnitzii* in the human gut [27]. Thus, FOS are broadly applicable prebiotic ingredients and potential supporting substrates for the growth and metabolic activity of butyrate-producing bacteria.

Based on these considerations, 2′-FL and FOS may play complementary roles in butyrate production. 2′-FL contributes to the supply of microbial metabolites, such as acetate, through bifidobacterial utilization. In contrast, FOS may support the growth of butyrate-producing bacteria by serving as an available substrate, thereby potentially enhancing the overall butyrate production. These interactions between bifidobacteria and butyrate-producing bacteria, often referred to as cross-feeding, have been extensively investigated. In particular, co-culture studies using *B. bifidum* and *F. prausnitzii* have demonstrated that *B. bifidum* degrades HMOs and provides HMO-derived metabolites and degradation products that support the growth and metabolic activity of *F. prausnitzii* [28]. Although the importance of interactions between bifidobacteria and butyrate-producing bacteria has been recognized, it remains unclear how the relative availability of complementary substrates influences butyrate production. In particular, the quantitative contributions of different 2′-FL/FOS ratios to butyrate production have not been systematically evaluated.

In the present study, we hypothesized that the 2′-FL/FOS ratio influences butyrate production through interactions between bifidobacteria and butyrate-producing bacteria. To test this hypothesis, we used a two-species co-culture model of *B. bifidum* and *F. prausnitzii*. Various ratios of 2′-FL and FOS were added to the co-culture, and bacterial growth dynamics, changes in bacterial abundance ratios, and SCFA production were evaluated at the 24 h endpoint. This study provides proof-of-concept evidence that in a simplified co-culture model, balancing complementary prebiotic substrates can regulate butyrate production in a ratio-dependent manner and may inform future studies on prebiotic formulation design.

## 2. Materials and Methods

### 2.1. Chemicals and Bacterial Strains

Unless otherwise stated, all chemical reagents were purchased from FUJIFILM Wako Pure Chemical Corporation (Osaka, Japan). Bacto^TM^ Tryptone, Bacto^TM^ Yeast Extract, and Difco^TM^ Agar were purchased from BD Difco (Franklin Lakes, NJ, USA). Vitamin B12 was obtained from Nacalai Tesque, Inc. (Kyoto, Japan). 2′-FL was obtained from Kyowa Hakko Bio Co., Ltd. (Tokyo, Japan). FOS was obtained from the FUJIFILM Wako Pure Chemical Corporation. According to the manufacturer’s instructions, the FOS preparation consisted of 1-kestose, nystose, and 1-fructofuranosylnystose. *Faecalibacterium prausnitzii* A2-165 (JCM31915) and *Bifidobacterium bifidum* JCM1254 were provided by the Japan Collection of Microorganisms, RIKEN BioResource Research Center, which participated in the National BioResource Project of the Ministry of Education, Culture, Sports, Science and Technology, Japan (Tsukuba, Japan).

### 2.2. Growth Experiments and Co-Culture Experiments

*F. prausnitzii* and *B. bifidum* were initially plated on yeast extract-casitone-fatty acid agar (YCFA) [29] supplemented with 0.5% (*w*/*v*) glucose as the sole energy source and incubated anaerobically at 37 °C for 24 h in a Whitley A25 Workstation (Don Whitley Scientific, Bingley, UK). Single colonies were inoculated into 1 mL of YCFA broth and incubated for 24 h. Cells were harvested by centrifugation at 6000× *g* for 5 min at 20 °C and resuspended in YCFA broth lacking sugars and volatile fatty acids (YCFA−). The bacterial suspension of each species was adjusted to an optical density at 600 nm (OD_600_) of 1.0 using a CO7500 Colorimeter (Biochrom Ltd., Cambridge, UK). Subsequently, 8 μL of each bacterial suspension was inoculated into 800 μL of YCFA− supplemented with 0.5% (*w*/*v*) carbohydrate in 48-well plates. For co-culture experiments, 8 μL of the OD_600_-adjusted suspension of each species was added to the same well, whereas monocultures were inoculated with 8 μL of the respective strain suspension alone. Thus, the initial bacterial density was standardized by adjusting each bacterial suspension to OD_600_ = 1.0 across all conditions. The carbohydrates tested were 2′-FL and FOS. For the combination treatments, 2′-FL and FOS were mixed at various weight ratios while maintaining a total carbohydrate concentration of 0.5% (*w*/*v*). Cultures were prepared either as co-cultures containing both strains or as monocultures containing either *F. prausnitzii* or *B. bifidum*. After anaerobic incubation at 37 °C for 24 h, OD_600_ was measured. The cultures were then centrifuged at 15,000 rpm for 5 min at 4 °C, and the supernatants and cell pellets were collected separately for SCFA analysis and DNA extraction, respectively. The supernatants and cell pellets were stored at −20 °C until analysis.

### 2.3. Quantitative Real-Time PCR

The relative abundance of *F. prausnitzii* and *B. bifidum* in the co-culture was quantified using qPCR. Bacterial cells were pelleted by centrifugation at 15,000 rpm for 5 min at 4 °C and stored at −20 °C until DNA extraction. Genomic DNA was extracted using ISOSPIN Fecal DNA (NIPPON GENE Co., Ltd., Tokyo, Japan) according to the manufacturer’s instructions. Previously reported 16S rRNA gene-targeting primer sets were used: FPR-2F, 5′-GGAGGAAGAAGGTCTTCGG-3′, and Fprau645R, 5′-AATTCCGCCTACCTCTGCACT-3′, for *F. prausnitzii*, and BiBIF-1, 5′-CCACATGATCGCATGTGATTG-3′, and BiBIF-2, 5′-CCGAAGGCTTGCTCCCAAA-3′, for *B. bifidum* [30]. qPCR was performed using the TB Green Premix Ex Taq (Takara Bio Inc., Kusatsu, Japan) on a QuantStudio 3 Real-Time PCR System (Thermo Fisher Scientific, Waltham, MA, USA). The cycling conditions were as follows: initial denaturation at 95 °C for 30 s, followed by 40 cycles of 95 °C for 5 s, annealing at 55 °C for 30 s, and extension at 72 °C for 45 s. Species-specific standard curves were generated using serial dilutions of genomic DNA extracted from each strain. Melting curve analysis was performed to confirm the amplification specificity. The relative abundance of species in the co-culture was calculated from species-specific qPCR values and expressed as the proportion of the combined abundance of *F. prausnitzii* and *B. bifidum*.

### 2.4. Organic Acid Analysis

Culture supernatants were obtained by centrifugation at 15,000 rpm for 5 min at 4 °C and stored at −20 °C until analysis. Organic acids were derivatized with 2-nitrophenylhydrazine using a Short- and Long-Chain Fatty Acid Analysis Kit (YMC Co., Ltd., Kyoto, Japan), according to the manufacturer’s protocol, with 2-ethylbutyric acid as an internal standard. The derivatives were analyzed using an LC-20 Prominence Series system (Shimadzu Corporation, Kyoto, Japan) equipped with a YMC-Pack FA column (6.0 × 250 mm I.D.; YMC Co., Ltd.). The separation was performed using a modified YMC-Pack FA method. The mobile phase consisted of solvents A (acetonitrile:methanol:water at 30:16:54 [*v*/*v*/*v*]) and B (acetonitrile:methanol:water at 20:16:64 [*v*/*v*/*v*]). Water used to prepare the mobile phases was obtained using a Milli-Q purification system (Merck Millipore, Burlington, MA, USA). The pH of each mobile phase was adjusted to 4.4–4.6 with 0.01 M HCl. The gradient program was as follows: solvent B was maintained at 100% from 0 to 21 min, decreased linearly to 0% from 21 to 22 min, held at 0% from 22 to 50 min, and then returned to 100%. The column was equilibrated for 9 min before the next injection. The flow rate was set at 1.2 mL/min, the injection volume was 10 μL, and the column temperature was maintained at 50 °C. Fatty acid derivatives were detected by UV absorbance at 400 nm, and the peaks were integrated using LCsolution software, version 3.50 (Shimadzu Corporation). The concentrations of the organic acids were calculated from calibration curves generated using authentic standards.

### 2.5. Statistical Analysis

All values are expressed as mean ± SD from triplicate experiments unless otherwise stated. Statistical analyses were performed using a one-way analysis of variance (ANOVA), followed by the Tukey–Kramer test using BellCurve for Excel, version 4.09 (Social Survey Research Information Co., Ltd., Tokyo, Japan). Statistical significance was defined as *p* < 0.05. The results of the Tukey–Kramer test were presented using a compact letter display (CLD), in which groups sharing at least one letter were not significantly different.

## 3. Results

### 3.1. Effects of 2′-FL and FOS Mixtures on Growth and Organic Acid Production of F. prausnitzii

**Figure 1:**
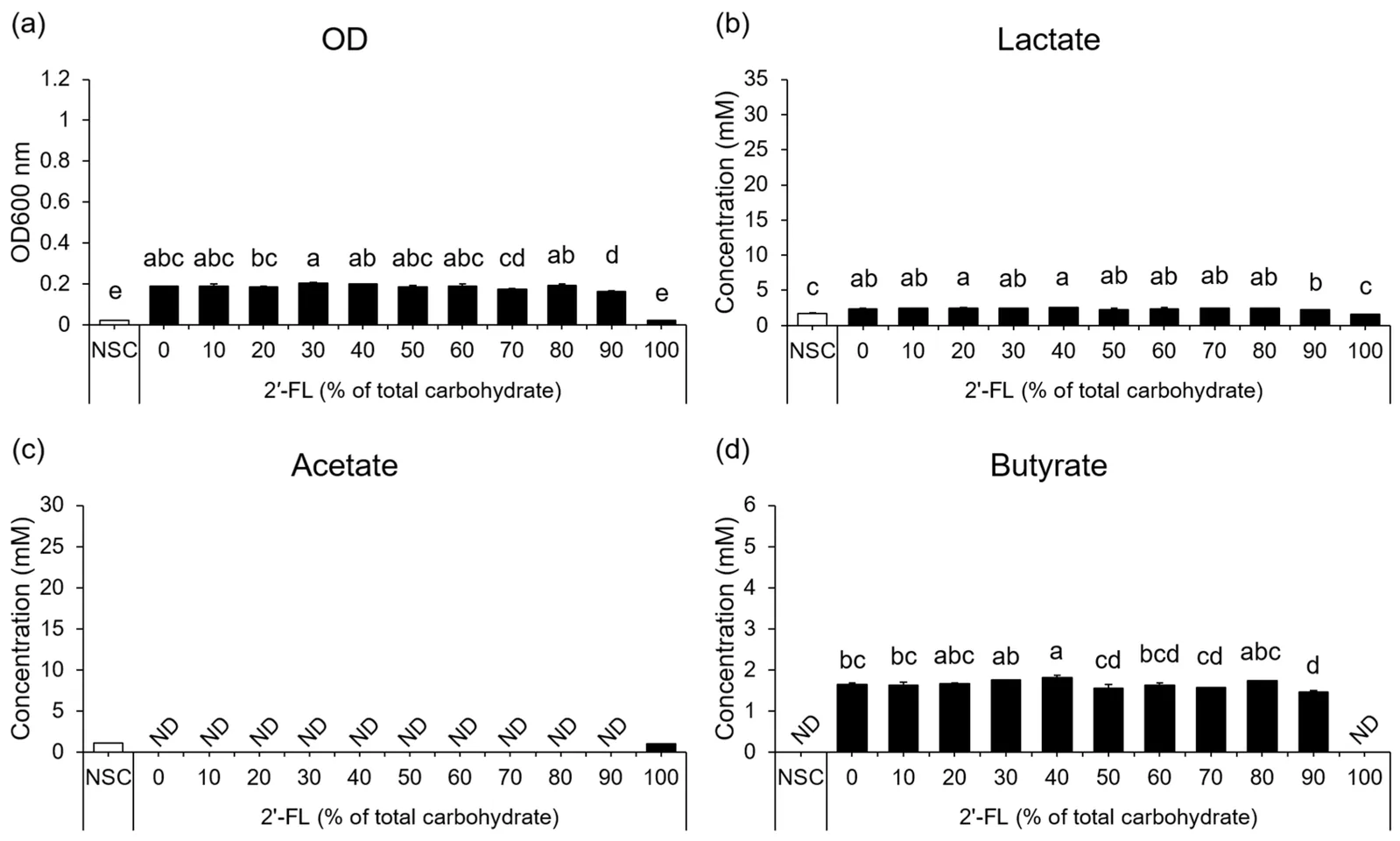
Growth and organic acid production of *F. prausnitzii* in monoculture under different 2′-FL/FOS substrate conditions. *F. prausnitzii* was cultured under the no-substrate condition (NSC) or in the presence of 2′-FL, FOS, or mixtures of 2′-FL and FOS. (**a**) Optical density at 600 nm (OD_600_) was measured after 24 h of incubation. Concentrations of (**b**) lactate, (**c**) acetate, and (**d**) butyrate in the culture supernatants were determined. For the 2′-FL, FOS, and mixed-substrate conditions, the total carbohydrate concentration was fixed at 5 g/L. Data are presented as means ± SD (*n* = 3). Statistical comparisons among the 2′-FL, FOS, and mixed-substrate conditions were performed using the Tukey–Kramer test. Significant differences are indicated by a compact letter display (CLD); groups sharing at least one letter are not significantly different, whereas groups with different letters are significantly different (*p* < 0.05). ND denotes values below the limit of detection (not detected).

*F. prausnitzii* did not grow when 2′-FL was provided as the sole carbohydrate source (100% 2′-FL). In contrast, growth was observed in media containing mixed substrates with 0–90% 2′-FL, corresponding to 100–10% FOS (Figure 1a). Acetate production was limited under all the conditions (Figure 1c). Lactate and butyrate were detected only under substrate conditions containing 0–90% 2′-FL, in which growth of *F. prausnitzii* was also observed (Figure 1b,d). Although these differences were statistically significant, absolute changes in lactate and butyrate production were small.

### 3.2. Effects of 2′-FL and FOS Mixtures on Growth and Organic Acid Production of B. bifidum

**Figure 2:**
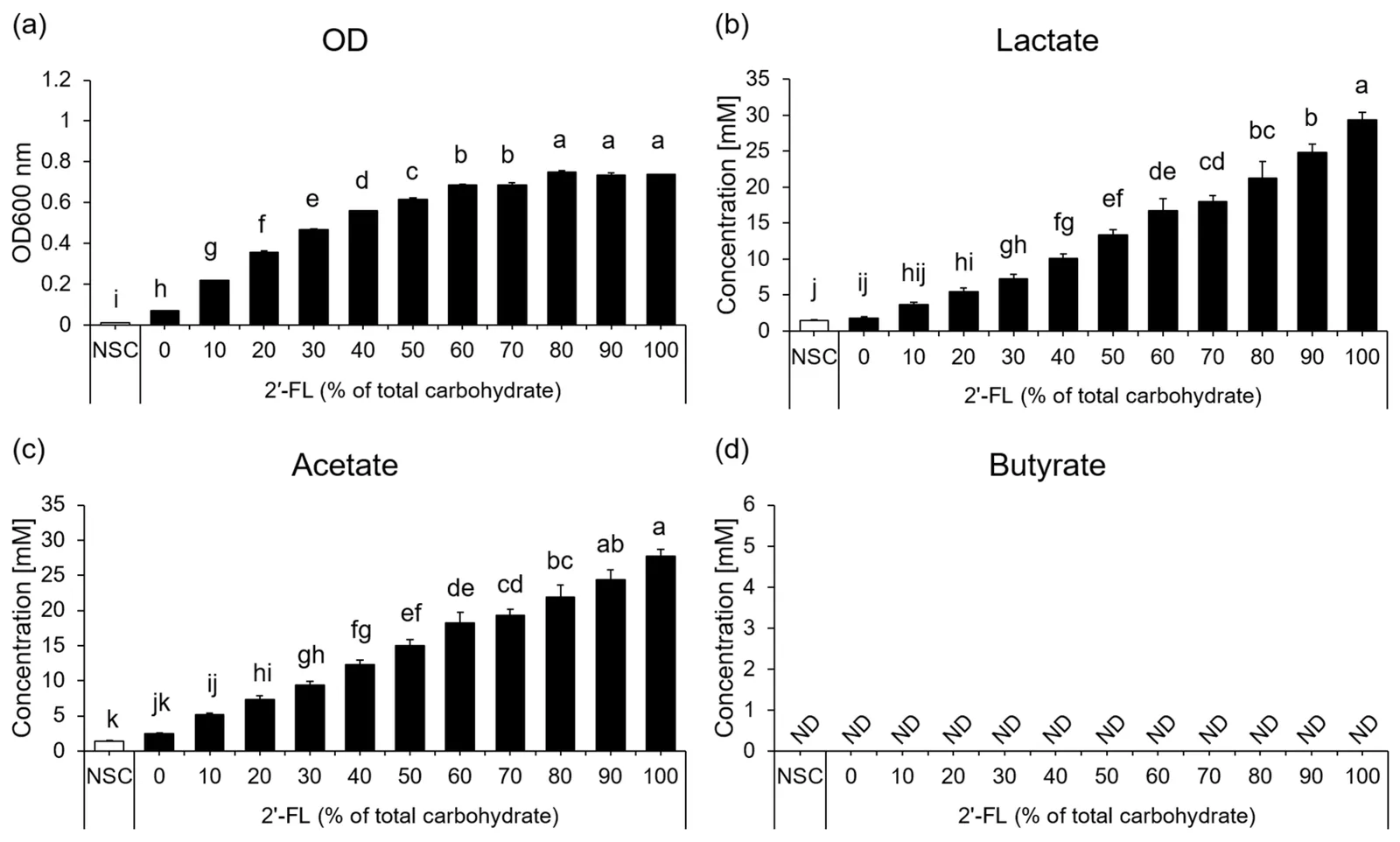
Growth and organic acid production of *B. bifidum* in monoculture under different 2′-FL/FOS substrate conditions. *B. bifidum* was cultured under the no-substrate condition (NSC) or in the presence of 2′-FL, FOS, or mixtures of 2′-FL and FOS. (**a**) Optical density at 600 nm (OD_600_) was measured after 24 h of incubation. Concentrations of (**b**) lactate, (**c**) acetate, and (**d**) butyrate in the culture supernatants were determined. For the 2′-FL, FOS, and mixed-substrate conditions, the total carbohydrate concentration was fixed at 5 g/L. Data are presented as means ± SD (*n* = 3). Statistical comparisons among the 2′-FL, FOS, and mixed-substrate conditions were performed using the Tukey–Kramer test. Significant differences are indicated by a compact letter display (CLD); groups sharing at least one letter are not significantly different, whereas groups with different letters are significantly different (*p* < 0.05). ND denotes values below the limit of detection (not detected).

The growth of *B. bifidum* increased progressively as the proportion of 2′-FL in the substrate mixture increased (Figure 2a). Lactate and acetate production showed a similar trend, increasing with the proportion of 2′-FL and reaching maximal levels under 100% 2′-FL conditions (Figure 2b,c). In contrast, butyrate production was not detected under any of the tested conditions (Figure 2d).

### 3.3. Effects of 2′-FL and FOS Mixtures on Growth and Organic Acid Production in a Co-Culture of F. prausnitzii and B. bifidum

**Figure 3:**
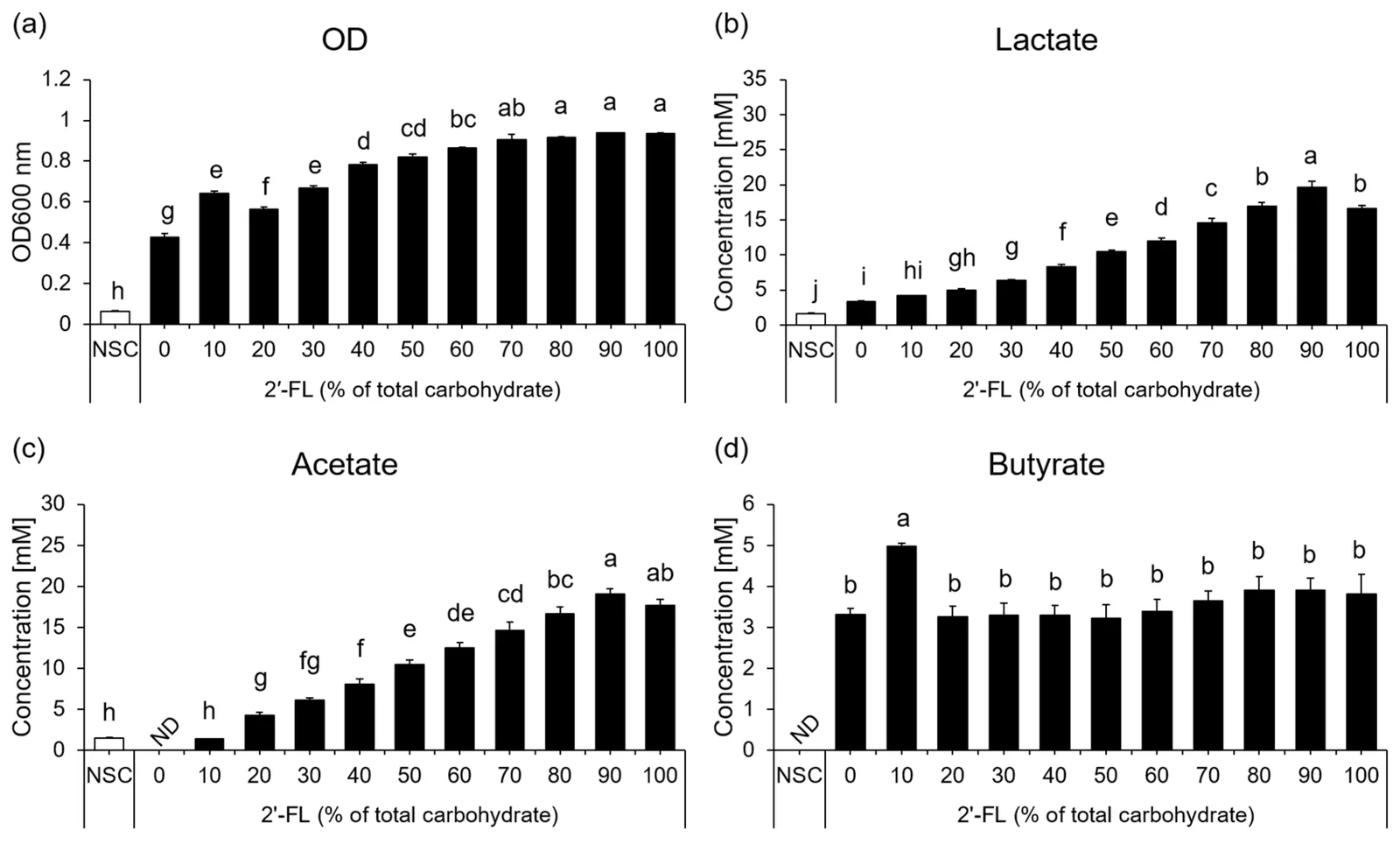
Growth and organic acid production in a co-culture of *F. prausnitzii* and *B. bifidum* under different 2′-FL/FOS substrate conditions.*F. prausnitzii* and *B. bifidum* were cultured under the no-substrate condition (NSC) or in the presence of 2′-FL, FOS, or mixtures of 2′-FL and FOS. (**a**) Optical density at 600 nm (OD_600_) was measured after 24 h of incubation. Concentrations of (**b**) lactate, (**c**) acetate, and (**d**) butyrate in the culture supernatants were determined. For the 2′-FL, FOS, and mixed-substrate conditions, the total carbohydrate concentration was fixed at 5 g/L. Data are presented as means ± SD (*n* = 3). Statistical comparisons among the 2′-FL, FOS, and mixed-substrate conditions were performed using the Tukey–Kramer test. Significant differences are indicated by a compact letter display (CLD); groups sharing at least one letter are not significantly different, whereas groups with different letters are significantly different (*p* < 0.05). ND denotes values below the limit of detection (not detected).

The total OD_600_ increased with increasing proportions of 2′-FL, with the highest values observed under 2′-FL-rich conditions, particularly at 80–100% 2′-FL (Figure 3a). Notably, the condition containing 10% 2′-FL and 90% FOS also showed a relatively elevated OD_600_ compared with the 0% and 20% 2′-FL conditions. Lactate and acetate production showed a similar trend, increasing with the proportion of 2′-FL and reaching their highest levels at 90% 2′-FL and 10% FOS (Figure 3b,c). In contrast, butyrate production was highest at 10% 2′-FL and 90% FOS and was significantly greater than that under the other tested conditions (Figure 3d).

### 3.4. Effects of 2′-FL and FOS Mixtures on the Relative Abundances of F. prausnitzii and B. bifidum in Co-Culture

**Figure 4:**
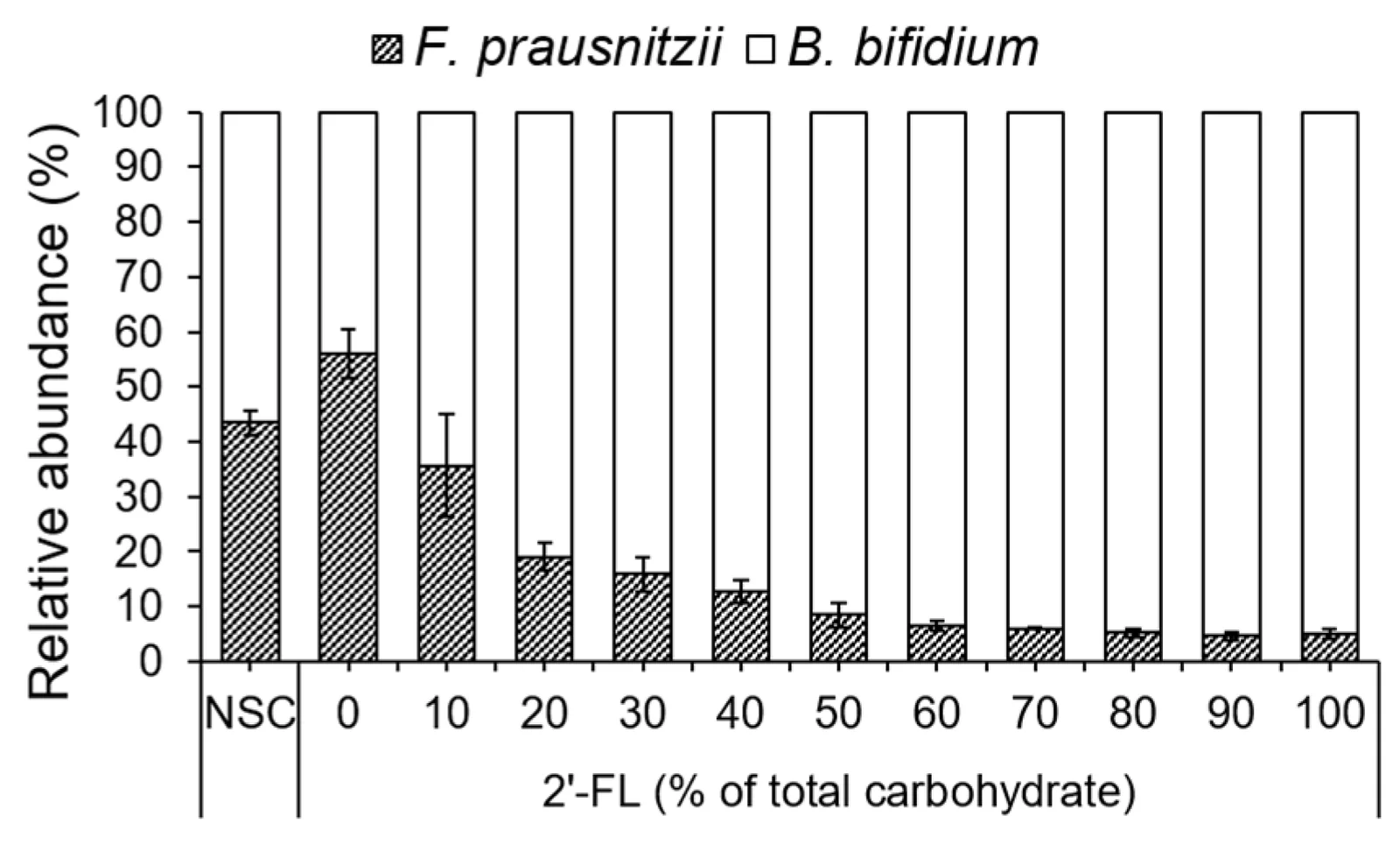
Relative abundances of *F. prausnitzii* and *B. bifidum* in co-culture under different 2′-FL/FOS substrate conditions. *F. prausnitzii* and *B. bifidum* were cultured for 24 h under a no-substrate condition (NSC) or in the presence of 2′-FL, FOS, or mixtures of 2′-FL and FOS. The relative abundances of *F. prausnitzii* and *B. bifidum* were quantified by quantitative PCR (qPCR). Bars represent *F. prausnitzii* (hatched) and *B. bifidum* (white). Data are presented as means ± SD (*n* = 3).

The relative abundances of *F. prausnitzii* and *B. bifidum* in the co-culture varied depending on the ratio of 2′-FL to FOS (Figure 4). Under the FOS-only conditions, *F. prausnitzii* accounted for approximately 60% of the total population. As the proportion of 2′-FL increased, *B. bifidum* became increasingly dominant, reaching a maximum relative abundance of 95%. Accordingly, the relative abundance of *F. prausnitzii* decreases as the proportion of 2′-FL increases. Species-specific genome copy numbers estimated from qPCR data are shown in Figure S1. The genome copy number of *F. prausnitzii* decreased under conditions containing 50% or more 2′-FL. In contrast, the genome copy number of *B. bifidum* increased with increasing proportions of 2′-FL and reached its highest value under the 60% 2′-FL condition.

## 4. Discussion

The present study tested the hypothesis that the combination of 2′-FL and FOS supports both the acetate supply from bifidobacteria and the growth and metabolic activity of butyrate-producing bacteria, thereby enhancing butyrate production through complementary microbial functions and possible metabolite-mediated interactions. To verify this, 2′-FL and FOS were mixed at different ratios and the effects of substrate balance on acetate supply and butyrate production were evaluated under two-species co-culture conditions.

Monoculture experiments revealed distinct substrate responses in *F. prausnitzii* and *B. bifidum*. *F. prausnitzii* grew in the presence of FOS-containing substrates; however, its growth was limited when 2′-FL was provided as the sole carbohydrate source. This finding suggests that, under the present experimental conditions, *F. prausnitzii* did not efficiently utilize 2′-FL as a primary carbon source, in line with previous reports showing limited growth of *F. prausnitzii* on HMOs [23,26,31,32]. In contrast, *B. bifidum* showed a stronger growth response as the proportion of 2′-FL increased, supporting earlier studies reporting that *B. bifidum* can degrade fucosylated oligosaccharides [28,33,34].

Under co-culture conditions, the total OD_600_ generally increased as the proportion of 2′-FL increased, broadly resembling the growth pattern observed in *B. bifidum* monoculture. This overall trend suggests that total co-culture growth was largely influenced by the growth response of *B. bifidum* to 2′-FL. However, the 10% 2′-FL and 90% FOS condition showed a locally elevated OD_600_ compared to the 0% and 20% 2′-FL conditions, although the maximum OD_600_ values were observed under 2′-FL-rich conditions. This deviation at a specific mixing ratio suggests that the co-culture exhibited growth behavior that could not be fully predicted from the monoculture profiles. This behavior may reflect changes in substrate utilization, metabolite exchange, or interspecies interactions between *B. bifidum* and *F. prausnitzii*.

Organic acid analysis further indicated that bacterial growth and butyrate production are not directly coupled. Under co-culture conditions, lactate and acetate production increased progressively as the proportion of 2′-FL increased, likely reflecting the growth and fermentation activities of *B. bifidum*. In contrast, butyrate production did not increase monotonically with either the proportion of 2′-FL or the total OD_600_. Instead, the highest butyrate production was observed under the 10% 2′-FL and 90% FOS condition, whereas the highest total OD_600_ values were observed under 2′-FL-rich conditions. This discrepancy suggests that the conditions that maximize overall bacterial growth differ from those that maximize butyrate production. This observation is important for interpreting our findings. If butyrate production had been determined simply by total bacterial biomass or by the extent of *B. bifidum*-derived acetate production, higher butyrate levels would have been expected under 2′-FL-rich conditions, where total OD_600_, lactate production, and acetate production were relatively high. Contrary to our expectations, a peak in butyrate production was observed in the 10% 2′-FL and 90% FOS condition. This suggests that efficient butyrate formation requires an appropriate balance between the metabolite-supplying activity of *B. bifidum* and the metabolite-converting capacity of *F. prausnitzii*. Thus, the 10% 2′-FL and 90% FOS condition may have provided a substrate environment in which acetate and other fermentation products supplied by *B. bifidum* were sufficiently available. In contrast, the growth and metabolic activity of *F. prausnitzii* were maintained under FOS-rich conditions.

Relative abundance analysis provided an additional perspective on butyrate production profiles. As the proportion of 2′-FL increased, *B. bifidum* became increasingly dominant, whereas the relative abundance of *F. prausnitzii* markedly decreased. Therefore, the decrease in butyrate production observed with increasing proportions of 2′-FL may be partly attributed to the decreased abundance of *F. prausnitzii*. A similar trend was observed for the genome copy numbers. The genome copy number of *F. prausnitzii* decreased with higher proportions of 2′-FL, whereas the genome copy number of *B. bifidum* increased with increasing proportions of 2′-FL. These observations support the view that substrate composition influences not only the metabolic activity but also the population structure of the co-culture. In other words, the butyrate production profiles observed across different substrate mixing ratios may reflect not only changes in metabolic interactions between the two bacteria, but also shifts in bacterial composition. However, the peak in butyrate production observed with the 10% 2′-FL condition cannot be fully explained by the simple assumption that it depends on the abundance of *F. prausnitzii*. Although the abundance of *F. prausnitzii* likely contributed to the observed butyrate production profile, the highest butyrate production was not observed under the conditions with the highest *F. prausnitzii* genome copy number. Rather, the enhanced butyrate production under these conditions was likely attributable to the coexistence of a sufficient *F. prausnitzii* population responsible for butyrate production and an adequate supply of metabolites from *B. bifidum*. Together, these findings suggest that complementary substrate combinations influence butyrate production by affecting both bacterial composition and metabolic interactions.

This interpretation is consistent with previous biochemical studies showing that metabolites supplied by acetate-producing bacteria contribute to butyrate production by *F. prausnitzii*. Duncan et al. reported that *F. prausnitzii* and several related butyrate-producing bacteria possess butyryl-CoA:acetate CoA-transferase activity and exhibit net acetate utilization during growth on glucose [18]. Stable isotope-based analyses have also shown that exogenous acetate can contribute substantially to the carbon content of butyrate in *F. prausnitzii* and *Roseburia* spp. [35]. These findings support the hypothesis that acetate supplied by acetate-producing bacteria can be incorporated into butyrate via cross-feeding. Therefore, in the present co-culture, acetate and possibly other fermentation products derived from *B. bifidum* may have contributed to butyrate formation by *F. prausnitzii* when the growth and metabolic activity of *F. prausnitzii* were sufficiently supported. Taken together, these findings suggest that FOS and 2′-FL play complementary but ratio-dependent roles in co-culture. FOS likely supported the growth and metabolic activity of *F. prausnitzii*, whereas 2′-FL enhanced the fermentation activity of *B. bifidum*, including acetate production. When the substrate balance shifted excessively toward 2′-FL, *B. bifidum* may have gained a competitive advantage, which may have reduced the abundance of *F. prausnitzii*. Consequently, the overall butyrate production may have been constrained despite the availability of acetate, a key substrate for butyrate formation by *F. prausnitzii*. Under FOS-only conditions, the supply of bifidobacteria-derived metabolites may have been insufficient. Thus, butyrate production may depend on metabolite availability, including that of acetate, and the abundance of butyrate-producing bacteria. Under the 10% 2′-FL and 90% FOS condition, this trade-off may have been alleviated by providing a sufficient metabolite supply from *B. bifidum* and maintaining an adequate population of *F. prausnitzii* responsible for butyrate production. This supply–conversion balance may explain why butyrate production was maximized independently of total co-culture growth.

This study had several limitations that should be acknowledged when interpreting our findings. First, bacterial abundance and metabolite production were evaluated only at the 24 h endpoint using a simplified two-species co-culture model. Although this approach is useful for investigating cross-feeding interactions, it does not fully reflect the temporal dynamics or ecological complexity of the human gut microbiota. In particular, although an optimal 2′-FL/FOS ratio was identified in this batch co-culture system, its applicability to the in vivo intestinal environment remains to be established, as substrate availability is influenced by continuous flow, host absorption, spatial heterogeneity, and interactions among diverse microbial populations. Future studies using continuous fecal fermentation models, time-course analyses, and human intervention trials are needed to evaluate whether the substrate-dependent butyrate production observed in this study is maintained under conditions that closely resemble the intestinal environment [36,37]. Second, the culture pH was neither measured nor controlled. Because acetate and lactate accumulated under 2′-FL-rich conditions, acidification may have contributed to the reduced abundance of *F. prausnitzii* and lower butyrate production observed under these conditions. Consequently, because the pH was not monitored, the extent to which the observed substrate ratio-dependent variation in butyrate production was driven by substrate availability itself or by pH-related changes in the culture environment resulting from organic acid accumulation remains unclear. Third, residual 2′-FL and FOS concentrations after 24 h were not quantified. Therefore, the contribution of substrate depletion, particularly under the 10% 2′-FL and FOS condition, to the observed metabolic profile remains unclear. Future studies should incorporate time-course measurements of residual substrates together with organic acid production, as bacterial abundance may provide a more comprehensive understanding of the metabolic dynamics underlying butyrate production. Fourth, the metabolic flux was not directly measured in this study. Future studies using stable isotope-labeled substrates, such as ^13^C-labeled acetate or ^13^C-labeled 2′-FL, are needed to determine whether increased butyrate production under 10% 2′-FL conditions reflects enhanced acetate uptake, increased butyrate pathway activity, or improved conversion efficiency by *F. prausnitzii*. Fifth, only one strain each of *B. bifidum* and *F. prausnitzii* was evaluated in this study. Because carbohydrate utilization pathways and butyrate-producing capacity can vary among strains, even within the same species, the optimal 2′-FL/FOS ratio may differ depending on strain-specific metabolic characteristics. For example, differences in the glycoside hydrolase repertoire involved in oligosaccharide degradation and the efficiency of acetate-dependent butyrate synthesis pathways can influence substrate utilization and cross-feeding interactions. Future studies should evaluate multiple strains and species to confirm the broad applicability of these findings.

## 5. Conclusions

In conclusion, using a minimal two-species co-culture model, this study showed that the mixing ratio of 2′-FL and FOS could nonlinearly regulate butyrate production through substrate complementarity and metabolite-mediated interactions between *B. bifidum* and *F. prausnitzii*. Among the tested conditions, a 2′-FL/FOS ratio of 10:90 (*w*/*w*) was the most effective for enhancing butyrate production, whereas conditions that maximized total co-culture growth did not necessarily maximize butyrate production. Although further studies are required to establish the translational relevance of these findings in vivo, our results provide valuable insights for the future design of prebiotic formulations.

## Data Availability

The original contributions presented in this study are included in the article/Supplementary Material. Further inquiries can be directed to the corresponding author.

## Supplementary Materials

The following supporting information can be downloaded at https://www.mdpi.com/article/10.3390/microorganisms14081812/s1, Figure S1: Estimated genome copy numbers of *F. prausnitzii* and *B. bifidum* in co-culture under different 2′-FL/FOS substrate conditions.

## Abbreviations

The following abbreviations are used in this manuscript:

2′-FL	2′-fucosyllactose
ANOVA	analysis of variance
CLD	compact letter display
FOS	fructooligosaccharides
HMOs	human milk oligosaccharides
NSC	no-substrate condition
qPCR	quantitative PCR
SCFAs	short-chain fatty acids
SD	standard deviation
YCFA	yeast extract-casitone-fatty acid agar
