# Supplementary material for: Ratio-Dependent Regulation of Butyrate Production by 2′-Fucosyllactose and Fructooligosaccharides in a Bifidobacterium bifidum-Faecalibacterium prausnitzii Co-Culture
Source: Microorganisms. 2026 Aug 17;14(8):1812. doi: 10.3390/microorganisms14081812 (PMC13515949; doi:10.3390/microorganisms14081812)
Supplement: Supplementary file 1 [file microorganisms-14-01812-s001.zip › microorganisms-4441586_SupplementaryData.pdf]

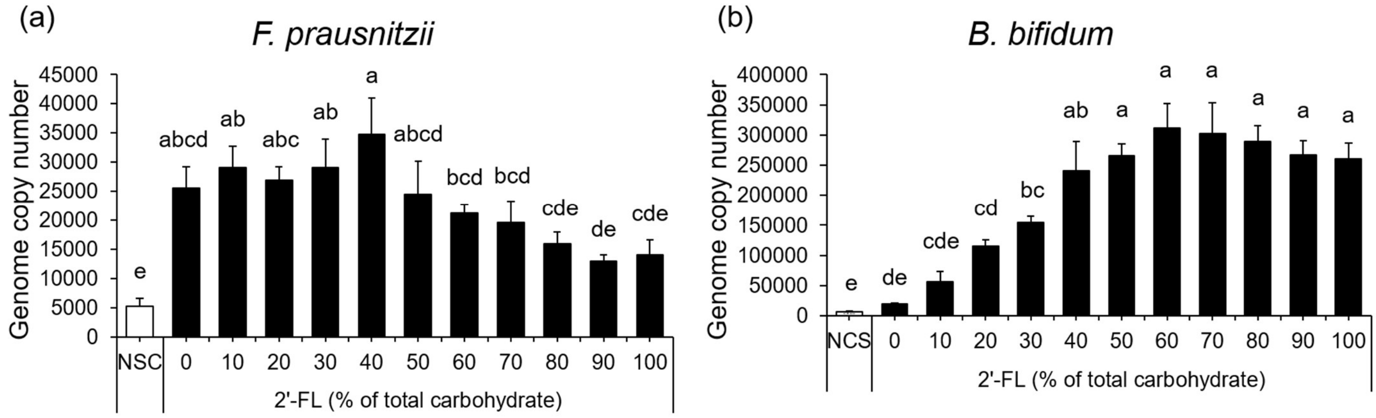

Supplementary Figure S1. Estimated genome copy numbers of *F. prausnitzii* and *B. bifidum* in co-culture under different 2′-FL/FOS substrate conditions. *F. prausnitzii* and *B. bifidum* were cultured for 24 h under a no-substrate condition (NSC) or in the presence of 2′-FL, FOS, or mixtures of 2′-FL and FOS. Genome copy numbers of (a) *F. prausnitzii* and (b) *B. bifidum* in the harvested cultures were quantified by quantitative PCR (qPCR). Data are presented as means ± SD (n = 3). Statistical comparisons among the 2′-FL, FOS, and mixed-substrate conditions were performed using the Tukey-Kramer test. Significant differences are indicated by a compact letter display (CLD); groups sharing at least one letter are not significantly different, whereas groups with different letters are significantly different ($p < 0.05$).
